# A Study on Optimal Strategy in Relative Radiometric Calibration for Optical Sensors

**DOI:** 10.3390/s17030490

**Published:** 2017-03-02

**Authors:** Kai Yu, Suhong Liu, Yongchao Zhao

**Affiliations:** 1 School of Geography, Beijing Normal University, Beijing 100875, China; yukai@mail.ie.ac.cn; 2 Key Laboratory of Technology in Geo-Spatial Information Processing and Application System, Institute of Electronics, Chinese Academy of Sciences, Beijing 100190, China; zhaoyc@mail.ie.ac.cn

**Keywords:** optical sensor, relative radiometric calibration, least square method

## Abstract

Based on the analysis of three main factors involved in the relative radiometric calibration for optical sensors, namely: the number of radiance level; the number of measurements at each level; and the radiance level grouping method, an optimal strategy is presented in this paper for relative radiometric calibration. First, the maximization to the possible extent of either the number of the radiance level or the number of measurements at each level can improve the precision of the calibration results, where the recommended number of measurements is no less than 20. Second, when the number of the radiance level is divisible by four, dividing all the levels evenly into four groups by intensity gradient order and conducting averages for each group could achieve calibration results with the highest precision, which is higher than the result of no grouping or any other grouping method with the mean square error being $2\sqrt{2}M_{n}/\sqrt{IT}$ (where $M_{n}$ is the mean square error of noise in the calibration data, I is the number of the radiance level, and T is the number of measurements for each level. In this case, the first two factors had an equivalent effect and showed their strongest effect on the precision. Third, when the calibration data were not evenly divided, the number of measurements demonstrated a stronger effect than the number of the radiance level. These cognitions are helping to achieve more precise relative radiometric calibration of optical sensors.

## 1. Introduction

In recent years, with the development of the sensor, data storage and transmission technology, the linear (or area) array imaging mode is widely used by optical Remote Sensing sensors, whose imaging quality and efficiency have also been significantly improved [1,2,3,4]. Subsequently, the problem of image radiation inconsistency caused by pixel response non-uniformity (PRNU) and noise interferences has become the focus of attention, which consists of relative radiometric calibration and correction. Relative radiometric calibration [5,6] aims to solve this problem by unifying the measurement results from various pixels to one certain reference standard in order to improve the quality of result data and minimize the impact of the PRNU on other applications using this data. The relative radiometric correction [7] eliminates the radiation inconsistencies of images with the relative radiometric calibration coefficients. The above-mentioned noise interferences include: stray light; the dark current; and stochastic noise, and can all viewed as synthetic noise. Stray light [8] is the diffuse radiation caused by the surroundings of the object and has positive correlation with incident radiance. The dark current [9] is the recorded value of the sensor’s internal current caused by electronic thermal motion when there is no incident radiation. The stochastic noise [10] is the unpredictable interference randomly generated during imaging.

The relationship between the energy received by the sensor and the measurement value (recorded by the sensor) can be expressed as Equation (1).

(1) B=u(R+S)+D+N

where B is the DN (digital number) value recorded by the sensor; u is the photoelectric response coefficient; R is the entrance pupil radiation; S is the stray light; D is the dark current; and N is the stochastic noise.

The existence of all these interfering factors has made the relative radiometric calibration a complex procedure where it has to measure multiple (more than three) objects (as the source of radiation) to get the data from all pixels. It then uses the least square fitting to obtain the linear transformation relationship between the reference standard and the data from each pixel, which is considered as the relative radiometric calibration coefficient. The effective minimization of the noise in the calibration data is the key to improving the precision of the relative radiometric calibration. If we use the data from pixel 0 as the reference standard, the relative radiometric calibration calculation for pixel *k* can be expressed as Equation (2).

(2) $$\begin{array}{l} \left[\begin{array}{cc} B_{k1} & 1\\ \vdots & \vdots \\ B_{kI} & 1 \end{array}\right]\left[\begin{array}{c} a_{k}\\ b_{k} \end{array}\right]=\left[\begin{array}{c} B_{01}\\ \vdots \\ B_{0I} \end{array}\right]\\ \left[\begin{array}{c} a_{k}\\ b_{k} \end{array}\right]=pinv(\left[\begin{array}{cc} B_{k1} & 1\\ \vdots & \vdots \\ B_{kI} & 1 \end{array}\right])\left[\begin{array}{c} B_{01}\\ \vdots \\ B_{0I} \end{array}\right] \end{array}$$

where B is the measured value with the first subscript being the pixel number and the second subscript being the radiation source number (for example, $B_{kI}$ represents the measured value for pixel k under the radiation source I); $a_{k}$ is the relative radiometric calibration gain coefficient and $b_{k}$ the offset coefficient for pixel k; and pinv is the function to compute the pseudo-inverse of a matrix.

When selecting a source of radiation, the standard source of radiation when calibration is conducted in labs is a tungsten lamp [11,12] or the sun [13,14,15,16,17]; while for in-orbit calibration, the artificial light source inside the sensor, or the sun, or the moon is selected as the source of radiation [18,19,20,21,22].

In general, homogeneous flat ground objects with stable reflectivity and good lambertian characteristics can be chosen as the target. For lab calibration, the integrating sphere or reference board is used as the target [23,24,25,26,27,28], while for in-orbit calibration, the internal integrating sphere or field areas such as White Sands in New Mexico; Rogers dry lake in California; Lunar Lake in Nevada; La Crau in Southern France; DunHuang; or QingHai Lake in China as targets [29].

In optimizing radiometric calibration, the most commonly used methods include increasing the number of radiance level for the target(s) (either the number of various radiation conditions for the same target or the number of various targets under the same radiation condition) before averaging the multiple measurements to reduce the noise interference; and to improve the precision of the radiometric calibration. For example, when calibration is conducted in the lab, measurement data is collected from the integrating sphere or reference board under various radiation conditions; for in-orbit calibration, measurements are conducted on various heterogeneous ground objects (the flat gobi or still water body) to obtain calibration data with different intensities; imagining patterns with a 90-degree yaw angle or continuously changing solar radiation are also used to acquire better quality calibration data [30,31].

**Figure 1:**
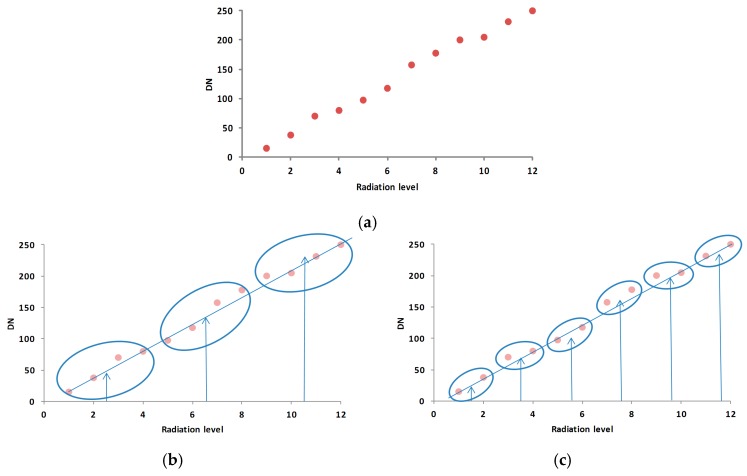
The schematic of grouping and its effect on fitting results. (**a**) scatter plot of pixel data; (**b**) data was divided into three groups; (**c**) data was divided into 6 groups. The horizontal axis and vertical axis are the radiation level and digital number (DN), respectively. The ellipses, arrows and oblique lines in (**b**,**c**) are grouping area, average results of each group and fitting results, respectively.

To summarize, there are three important elements for the relative radiometric calibration process: the number of radiance level; the number of measurements at each level; and when the above two elements are fixed, the way of dividing the acquired measurement data into groups before merging them. The grouping method we discussed in this paper is that averaging the multiple measurements of each level, and then dividing all the levels into groups by intensity gradient order and conducting averages for each group. Grouping controls the number of point pairs and, consequently, the accuracy of least square fits, as shown in Figure 1. A higher precision can be achieved through the adjustment of these three elements. This paper first quantitatively analyzes the impact these three elements have on the precision of the relative radiometric calibration from a theoretical perspective; second, it illustrates the conclusions using computer simulations; and finally outlines the strategy in achieving the optimized precision.

## 2. Materials and Methods

Supposing the sensor has K pixels, where the number of radiance level is I and the number of measurements conducted at each radiation level is T, then Equation (1) can be rewritten as Equation (3).

(3) $$B_{ki}=u_{k}(R_{i}+S_{ki})+D_{k}+N_{ki}$$

where $B_{ki}$ is the measurement result of pixel k at the radiation level i; $u_{k}$ is the photoelectric response coefficient of pixel k; $R_{i}$ is the absolute radiance at the entrance pupil of the pixel at the radiation level i; $D_{k}$ is the dark current of pixel k; $S_{ki}$ is the stray light of pixel k at the radiation level i; $N_{ki}$ is the corresponding stochastic noise of pixel k at the radiation level i with its mean square error being . $D_{k}$, $S_{ki}$, $N_{ki}$ can be combined as synthetical noise of pixel k, which usually follows Gaussian distribution. The following discussions are all based on the assumption of Gaussian noise.

The stray light received by pixel k is determined by the radiation intensity of the radiation source $R_{i}$ and the pixel’s position in the space at point $\left(x_{k},y_{k},z_{k}\right)$, where the space position can be defined as the stray light response $v_{k}$. With all this, Equation (3) can be transformed into Equation (4).

(4) $$\begin{array}{l} B_{ki}=u_{k}(R_{i}+v_{k}R_{i})+D_{k}+N_{ki}\\ \begin{array}{c} \begin{array}{l} \;=u_{k}(1+v_{k})R_{i}+D_{k}+N_{ki}\\ \;=u\prime_{k}R_{i}+D_{k}+N_{ki} \end{array} \end{array} \end{array}$$

where $u\prime_{k}$ is the synthesis of the photoelectric coefficient and the stray light response coefficient for pixel k which is called the synthetical radiation response coefficient.

The average result for all the measurements of T times at each level of radiation intensity can be expressed as Equation (5).

(5) $${\bar{B}}_{ki}=\frac{1}{T}\sum_{t=1}^{T}B_{ki}^{t}=\frac{1}{T}\sum_{t=1}^{T}\left(u\prime_{k}R_{i}+D_{k}+N_{ki}^{t}\right)=u\prime_{k}R_{i}+D_{k}+\bar{N_{ki}}$$

where the superscript t represents the data number; $\bar{N_{ki}}$ is the average value of the noises from all the measurements of T times with a value of $\frac{1}{T}\sum_{t=1}^{T}N_{ki}^{t}$. If compared with $B_{ki}$, the mean square error of ${\bar{B}}_{ki}$ is reduced to M′n=(1T2∑t=1TMn2)12=MnT−12.

It is supposed that all the measurement results obtained from all the I levels of radiation intensity were divided into J groups, $P_{1},P_{2},\cdots ,P_{J}$ ($\sum_{j=1}^{J}P_{j}=I$ and $P_{j}\geq 0$), where each group had the data from the corresponding radiance level and the data series in the groups were numbered as $c_{1},\cdots ,e_{1}$, $c_{2},\cdots ,e_{2}$, ……, and $c_{J},\cdots ,e_{J}$ respectively. An average for each group, represented as $A_{1},\cdots ,A_{J}$ respectively, was conducted in order to further reduce the noise interference which can be expressed as Equation (6).

(6) $$\{\begin{array}{c} A_{1}=\frac{1}{P_{1}}\sum_{l=c_{1}}^{e_{1}}\left(u\prime_{k}R_{l}+D_{k}+\bar{N_{kl}}\right)\\ \vdots \\ A_{J}=\frac{1}{P_{J}}\sum_{l=c_{J}}^{e_{J}}\left(u\prime_{k}R_{l}+D_{k}+\bar{N_{kl}}\right) \end{array}$$

The mean square errors of $A_{1},\cdots ,A_{J}$ were further reduced and can be expressed as Equation (7).

(7) {MA1=(1P12∑l=c1e1M′n2)12=Mn(TP1)−12⋮MAJ=(1PJ2∑l=cJeJM′n2)12=Mn(TPJ)−12

According to the error propagation theory, when the error of each element for the data series of reference pixel 0 is expressed as $E={\left[E_{k1},\cdots ,E_{kI}\right]}^{T}$ (where the right superscript T represents the matrix transposition operation), this error will be propagated to the radiometric calibration coefficients $a_{k}$ and $b_{k}$ during the process of using the least square method. Their mean square errors have the same value [32], which can be represented in Equation (8).

(8) Mo=±(ETEJ−L)12

where L=2 represents that there are two coefficients for the linear fitting in the relative radiometric calibration. Substituting the error turning Equation (7) into Equation (8), the mean square error M can be expressed as Equation (9).

(9) M=±(Mn2T−1P1−1+⋯+Mn2T−1PJ−1J−L)12=Mn(T−1∑j=1JPj−1J−L)12

Given the parameters I and T, there are two cases to be discussed to minimize the error, M.

**Case 1:**

When J is fixed, getting the minimum value of M is equivalent to minimizing $\sum_{j=1}^{J}P_{j}^{-1}$. Based on the am-gm inequality theorem [33], we have the following inequation in Equation (10).

(10) P1+⋯+PJ≥J(P1⋯PJ)1J=J1(1P1⋯PJ)1J≥J21(1P1+⋯+1PJ)

Based on Equation (10), the following inequation Equation (11) is derived.

(11) $$\frac{1}{P_{1}}+\cdots +\frac{1}{P_{J}}\geq \frac{J^{2}}{P_{1}+\cdots +P_{J}}$$

Therefore, when $P_{1}=\cdots =P_{J}$, the equality of Equation (11) holds, which produces the minimum value for M. It indicates that the even group-division will achieve a higher precision than any other grouping methods.

**Case 2:**

When J is not fixed, by substituting $P_{1}=\cdots =P_{J}=\frac{I}{J}$ into Equation (9), we have Equation (12).

(12) M=Mn(T−1∑j=1JPj−1J−L)12=Mn(T−1J2IJ−L)12=Mn(J2IT(J−L))12

By calculating the derivative of Equation (12) against J, we have Equation (13).

(13) ∂M∂J=∂(Mn(J2IT(J−L))12)∂J=12Mn(J2IT(J−L))−12J(J−2L)IT(J−L)2

From Equation (13), it can be concluded that when J=2L, M gets its minimum value, which indicates that it can achieve the highest precision when the data set is evenly divided into four groups and the minimum value of the error is expressed in Equation (14).

(14) $$M=2M_{n}\sqrt{\frac{L}{IT}}=\frac{2\sqrt{2}M_{n}}{\sqrt{IT}}$$

If no grouping is done, which means J=I and $P_{1}=\cdots =P_{J}=1$, the error has the following value in Equation (15).

(15) $$M=M_{n}\sqrt{\frac{I}{(I-L)T}}$$

**Figure 2:**
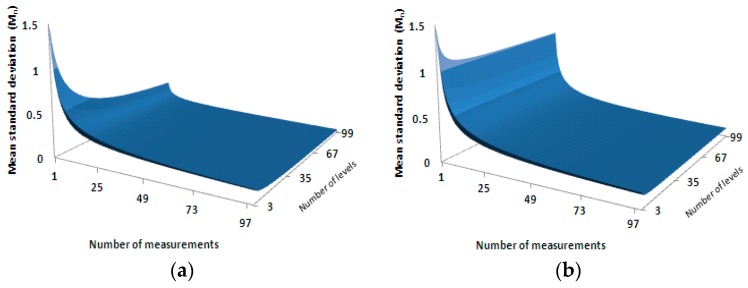
The relationship between the precision and the two parameters, the number of the radiance level and the number of measurements. The horizontal axes are the number of measurements and the number of radiance level, respectively, and the vertical axis is the precision (mean standard deviation) of the calibration result. (**a**) relationship surface with four evenly-divided groups; (**b**) relationship surface without grouping.

As seen from Equations (14) and (15), whether using the grouping strategy or not, the higher the number of the radiance level and the number of measurements at each level, the higher the precision becomes, i.e., the results of the relative radiometric calibration are much closer to the theoretical values without noise interference. When the radiance level I is fixed, precision will be improved if the number of measurements, T at each level is increased; when T is fixed, the precision will be improved as I is increased. Figure 2 illustrates the relationship between the precision and these two parameters, namely the number of the radiance level and the number of measurements at each radiation level, in a 3D surface manner where Figure 2a represents the case when the data is evenly divided into four groups and Figure 2b without the grouping. The two horizontal axes represent the number of the radiance level and the number of measurements at each level, respectively. The vertical axis represents the mean square error of the relative radiometric calibration coefficients.

As illustrated in Figure 2, in either case, the calibration precision was increased along with the increase of the number of the radiance level or the increase of the number of measurements at each level. When using the even-division grouping method to group the data from multiple radiance levels, the number of the radiance level and the number of measurements had the equivalent effect and showed their strongest effect when the grouping number was four (as shown in Equation (14)). As expressed by Equation (15), when no grouping was done, the number of measurements had a stronger impact than the number of the radiance level.

## 3. Results

To verify the theory and illustrate the conclusions, the simulation was conducted to cover three cases with the parameters defined in Table 1.

**Case 1:** the number of pixels, the number of the radiance level and the grouping method were all fixed; however, the number of measurements at each level was not fixed;

**Case 2:** the number of pixels, the grouping method and the number of measurements at each level were all fixed; however, the number of the radiance level was not fixed;

**Case 3:** the number of pixels, the number of the radiance level and the number of measurements at each level were all fixed, but the grouping method was not fixed.

The simulation data was quantified using two bytes with the valid value from 0–65,535. The maximum value of the stochastic noise was less than 5% of the maximum quantified value (3276). The multiplicative factor (synthetical radiation response coefficient) of each pixel was the sinusoidal values from 0.25 π to 0.75 π with 0.005 π being the interval. The additive factor (synthesis of dark current and stochastic noise) is random data with its maximum value less than 5% of the maximum quantified value. The gain coefficient is the reciprocal of the multiplicative factor and the offset coefficient is the quotient of the additive factor divided by the multiplicative factor. The radiation level was evenly distributed using the same interval in the range of 10%–90% of the maximum quantified value. Pixel 51 was selected as the reference standard.

**Case 1**: to understand the impact of the number of averaging measurements for each level on the precision.

**Figure 3:**
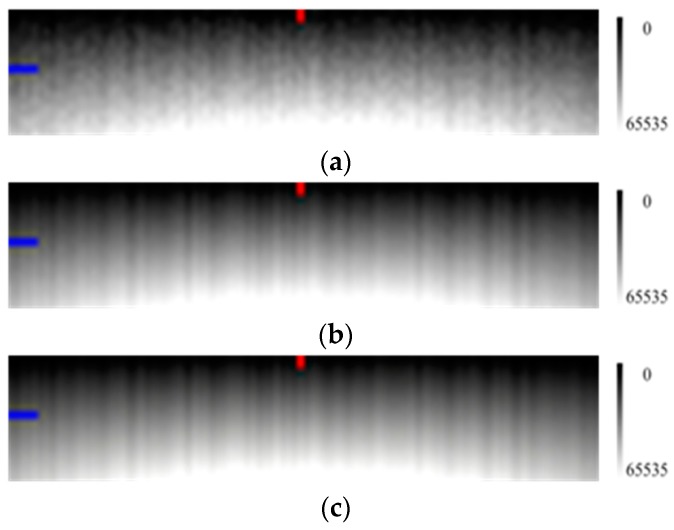
Simulated calibration images (100 samples × 20 lines) using various numbers of measurements with the horizontal direction for the pixels and the vertical direction for the radiance level (the radiation energy is gradually enhanced from the top to the bottom as the right grayscale). (**a**) measurement conducted once; (**b**) measurements conducted 20 times; and (**c**) measurements conducted 100 times. Column 50 and line 10 are respectively marked with red and blue labels.

In Figure 3, it is clear that when the number of measurements increases, the image sharpens and the radiation response difference among the pixels is more obvious.

**Figure 4:**
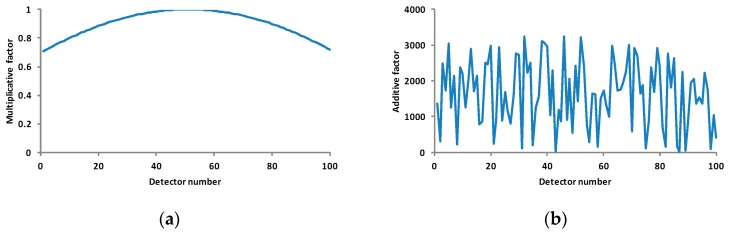

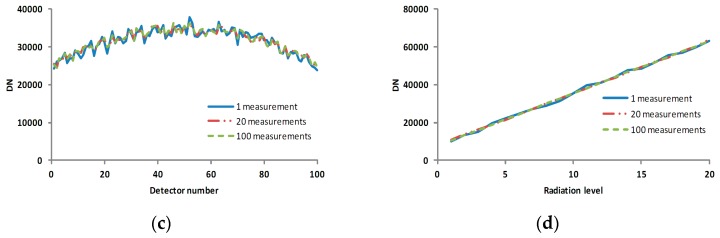
The measurement data profile with the multiplicative factor, the additive factor and various numbers of measurements. (**a**) the curve of multiplicative factors of pixels; (**b**) the curve of additive factors of pixels; (**c**) the profile of line 10 in Figure 3; and (**d**) the profile of column 50 in Figure 3. The horizontal axes of (**a**–**c**) are the pixel number, and the vertical axes of (**a**–**c**) are the multiplicative factor, additive factor and DN, respectively. The horizontal axis and vertical axis of (**d**) are the radiation level and DN, respectively.

Figure 4c,d also showed that increasing the number of measurements can reduce noise effect and make profiles more smooth.

**Figure 5:**
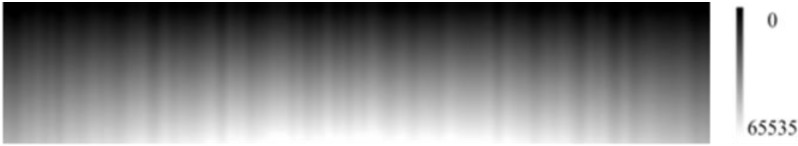
Simulated verification image (100 samples × 20 lines) with 20 times measurements.

Figure 5 is the verification image, whose multiplicative and additive factor are the same as that of the calibration image in Figure 3. Both images have noises of the same characteristic but different values.

**Figure 6:**
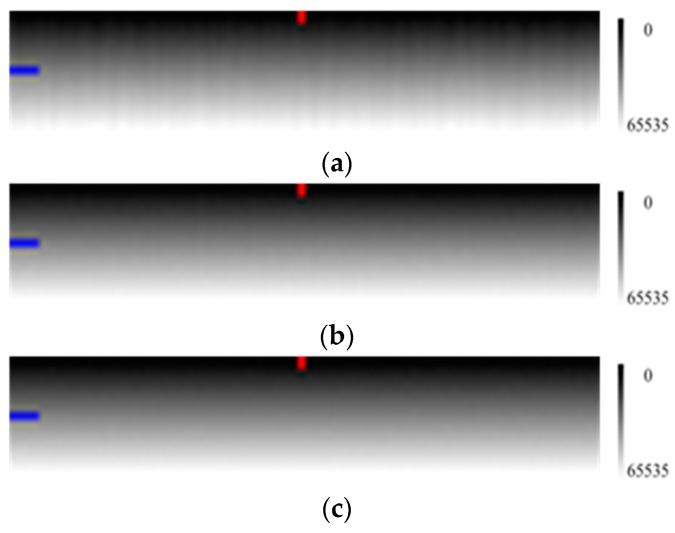
The relative calibration results of the verification image with the horizontal direction for the pixels and the vertical direction for the radiance level. (**a**) the result with the calibration coefficient from Figure 3a; (**b**) the result with the calibration coefficient from Figure 3b; and (**c**) the result with the calibration coefficient from Figure 3c. Column 50 and line 10 are marked with red and blue labels, respectively.

As shown in Figure 6, the relative calibration result was much better with the calibration coefficients from larger numbers of the averaged measurements.

**Figure 7:**
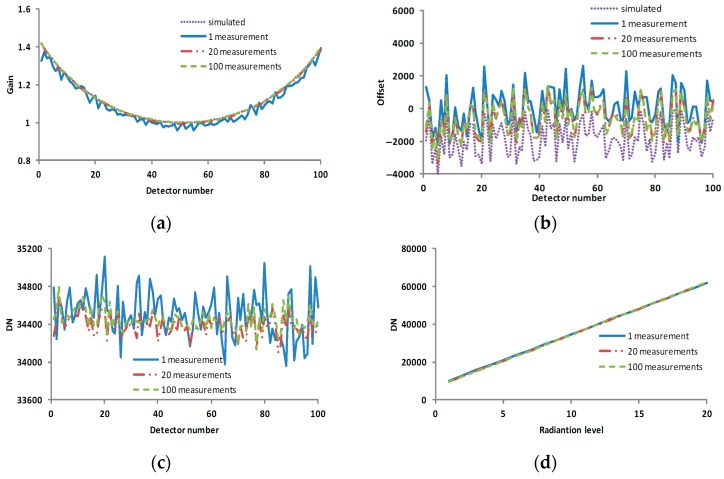
The profiles of the calibration coefficients and corrected results in various cases. (**a**) the gain coefficient in various scenarios; (**b**) the offset coefficient in various scenarios; (**c**) the profile of line 10 in the corrected result corresponding to Figure 4c; and (**d**) the profile of column 50 in the corrected result corresponding to Figure 4d. The horizontal axes of (**a**–**c**) are the pixel number, and the vertical axes of (**a**–**c**) are the gain coefficient, offset coefficient and DN, respectively. The horizontal axis and vertical axis of (**d**) are the radiation level and DN, respectively.

As seen in Figure 7, the calculated calibration coefficients became much closer to the simulated calibration coefficients as the number of measurements increased, and the corresponding corrected result was also improved.

**Figure 8:**
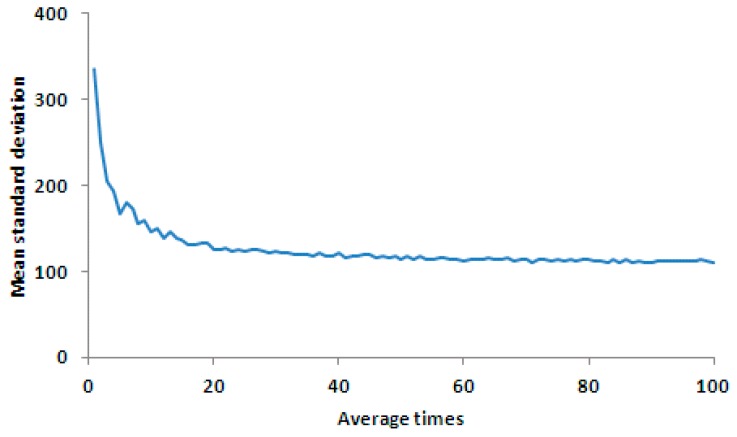
Mean value curve of the standard deviation of the corrected results for each line for various measurements. The horizontal axis is the average times, and the vertical axis is the precision of correction result (mean standard deviation).

As illustrated in Figure 8, the larger the number of averaging measurements, the smaller the standard deviation of the corrected result. The error was reduced dramatically when the number of averaging was increased from three to 20. When the number of averaging was larger than 20, the error was reduced more slowly.

**Case 2**: to understand the impact of the number of the radiance level on the precision.

**Figure 9:**
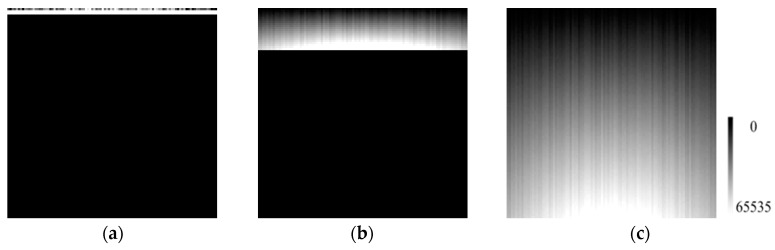
Simulated calibration data with different numbers of radiance level with the horizontal direction for the pixels and the vertical direction for the radiance level. The below black portions in (**a**,**b**) are filled with 0. (**a**) 3 radiance level; (**b**) 20 radiance level; and (**c**) 100 radiance level.

**Figure 10:**
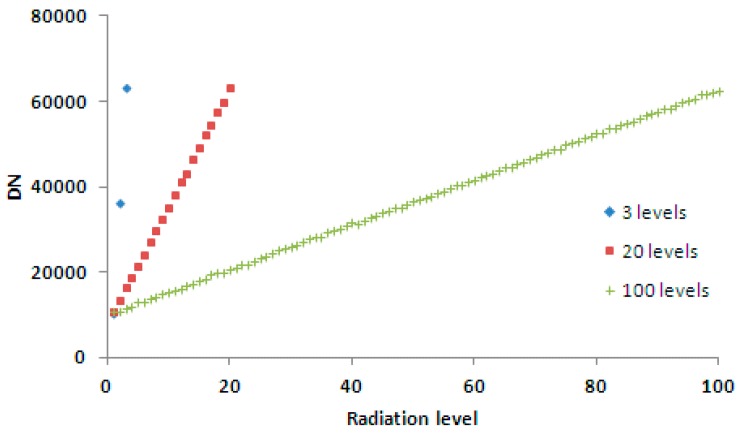
The vertical profile of the calibration data for column 50 corresponding to Figure 9. The horizontal axis is radiation level, and the vertical axis is DN.

As illustrated in Figure 9 and Figure 10, the numerical ranges of the columns with the same column number were duplicated, but the interval between levels was smaller as the number of levels increased.

**Figure 11:**
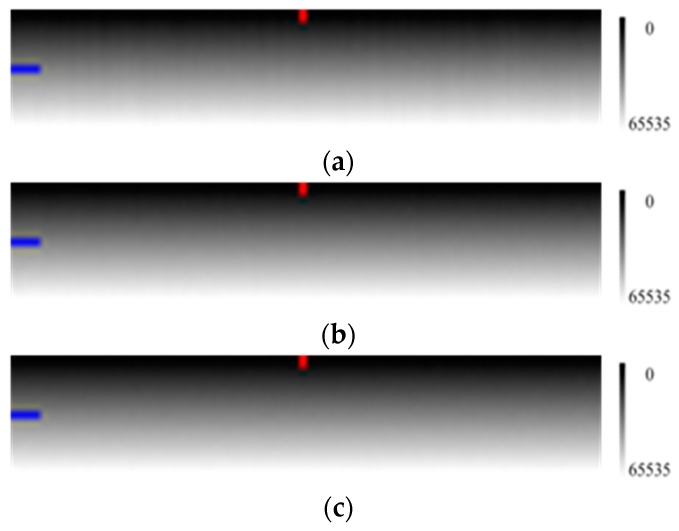
The relative calibration results of the verification image with gain and offset coefficients from a different number of radiance level, where the horizontal direction is the pixels and the vertical direction is radiance level. (**a**) the result with the coefficient from Figure 9a (3 radiance level data); (**b**) the result with coefficient from Figure 9b (20 radiance level data); and (**c**) the result with coefficient from Figure 9c (100 radiance level data). Column 50 and line 10 are marked with red and blue labels, respectively.

As shown in Figure 11, the relative calibration result was better with the calibration coefficients from larger numbers of the radiance level.

**Figure 12:**
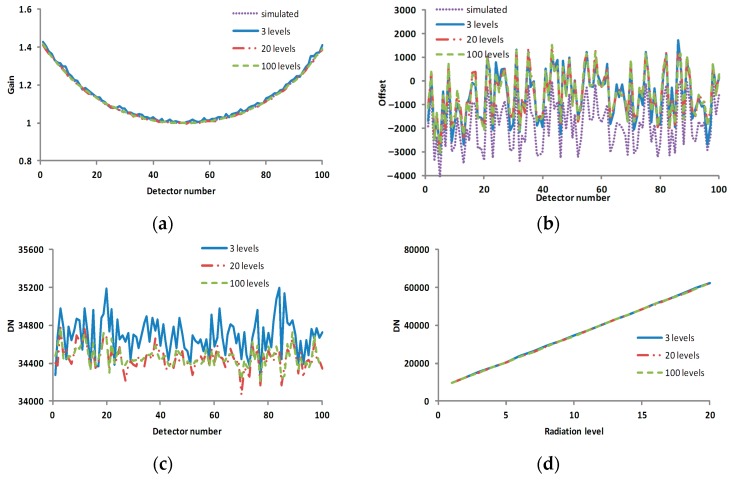
The profiles of the calibration coefficients and corrected results in the various scenarios of Case 2. (**a**) the gain coefficient in various scenarios; (**b**) the offset coefficient in various scenarios; (**c**) the profile of line 10 in the corrected result corresponding to Figure 11; and (**d**) the profile of column 50 in the corrected result corresponding to Figure 11. The horizontal axes of (**a**–**c**) are pixel number, and the vertical axes of (**a**–**c**) are the gain coefficient, offset coefficient and DN, respectively. The horizontal axis and vertical axis of (**d**) are radiation level and DN, respectively.

As seen from Figure 12, the calculated gain coefficients became much closer to the simulated gain coefficients and the calculated offset coefficients had little change as the number of levels increased, so the corresponding corrected result was improved.

**Figure 13:**
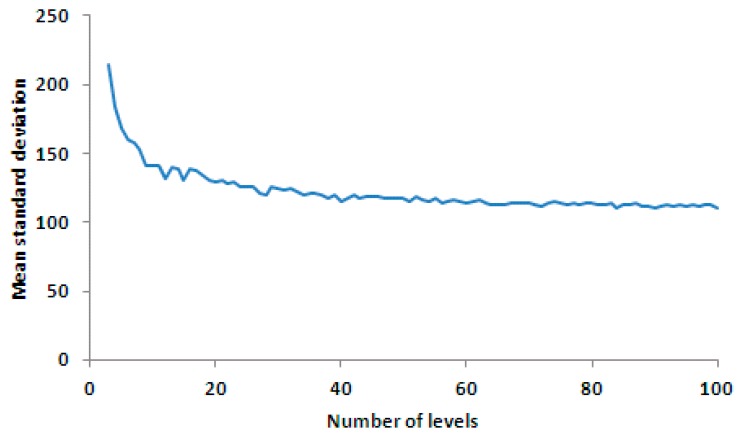
Mean value curve of the standard deviation of the corrected results for each line for various numbers of radiance level. The horizontal axis is the number of levels, and the vertical axis is the precision of correction result (mean standard deviation).

As illustrated in Figure 13, the bigger the number of radiance level, the smaller the standard deviation of the corrected result. The error was reduced dramatically when the number of radiance level was increased from three to 30. When the number of the radiance level was bigger than 30, the error was reduced more slowly, along with the increase of the number of the radiance level.

**Case 3**: to understand the impact of the grouping method on the precision.

**Figure 14:**
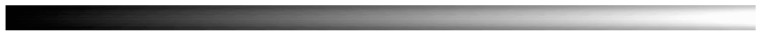
The image of the transposition of the simulated calibration data with 100 samples × 3000 lines (horizontal direction and vertical direction are reversed to that of Figure 3).

Figure 14 is the simulated calibration data for grouping method analysis.

**Figure 15:**
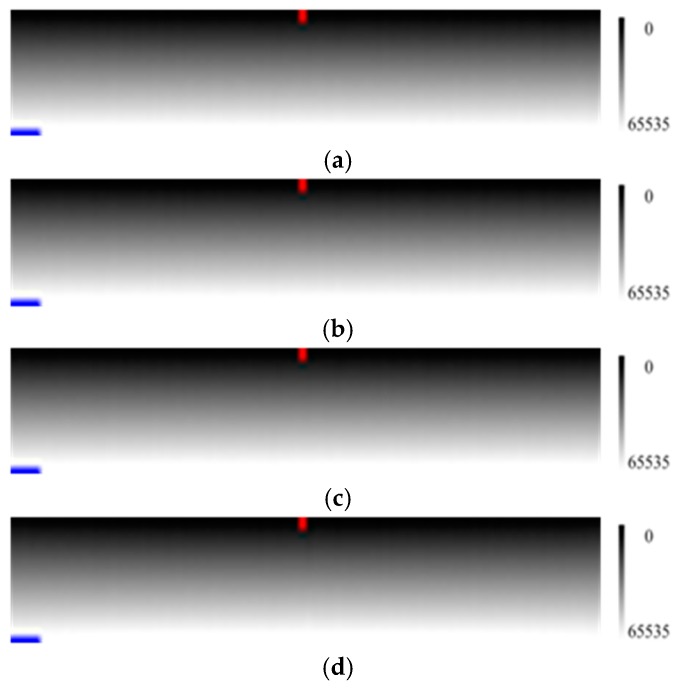
The relative calibration results of the verification image with gain coefficient and offset coefficient calculated from various grouping cases, where the horizontal direction is pixels and vertical direction is radiance level. (**a**) the result with coefficient from three groups case; (**b**) the result with coefficient from four groups case; (**c**) the result with coefficient from 500 groups case; and (**d**) the result with coefficient from 3000 groups case. Column 50 and line 20 are marked with red and blue labels, respectively.

We divided the 3000 radiance level evenly into 3, 4, …, 1500, 3000 groups, which was divisible to 3000, and conducted average calculation for each level and each group, followed by the calculation of the relative radiometric calibration coefficients using pixel 51 as the standard reference. Correcting the verification image by the calculated relative radiometric calibration coefficients of various grouping cases, we obtained the results shown in Figure 15.

**Figure 16:**
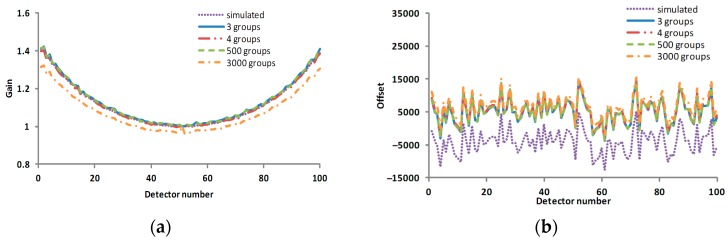

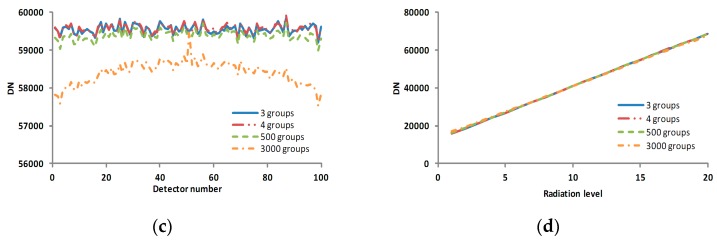
The profiles of the calibration coefficients and corrected results in the various scenarios of Case 3. (**a**) the gain coefficient in various scenarios; (**b**) the offset coefficient in various scenarios; (**c**) the profile of line 20 in the corrected result corresponding to Figure 15; and (**d**) the profile of column 50 in the corrected result corresponding to Figure 15. The horizontal axes of (**a**–**c**) are the pixel number, and the vertical axes of (**a**–**c**) are gain coefficient, offset coefficient and DN, respectively. The horizontal axis and vertical axis of (**d**) are radiation level and DN, respectively.

Although the differences among the corrected result images in Figure 15 are not obvious, it is still shown in Figure 16 that the calculated gain coefficients became much closer to the simulated gain coefficients and the calculated offset coefficients had little change as the number of groups decreased, and the corresponding corrected result was improved. The standard deviations of the profiles in Figure 16c for 3, 4, 500, 3000 groups are 122.73, 120.32, 129.53 and 302.57, respectively.

**Figure 17:**
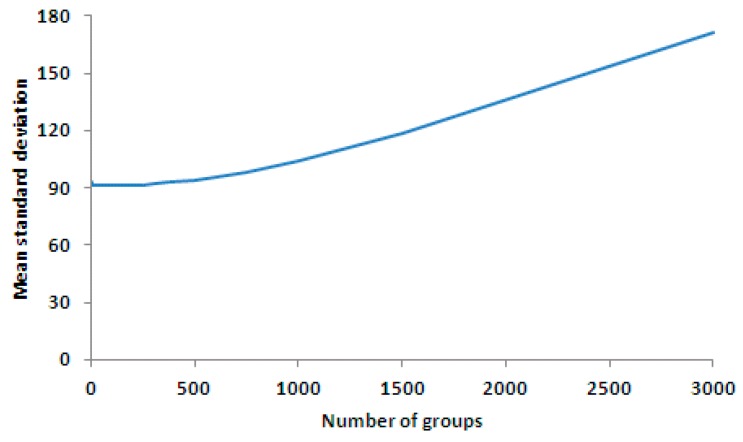
Mean value curve of the standard deviation of the corrected results for each line in various grouping cases. The smallest mean standard deviation is 91.02 at four groups. The horizontal axis is the number of groups, and the vertical axis is the precision of the correction result (mean standard deviation).

As illustrated by Figure 16 and Figure 17, the calibration result produced by four groups even-division was better than that of any other even-division. When the number of groups was increased, the calibration result became worse, which coincides with the conclusion from Equation (13).

## 4. Discussion

It was difficult to increase the radiance level for the targets during the course of the experiment when conducting the traditional relative radiometric calibration (no matter which method, either lab calibration, or in-orbit internal, or in-orbit synchronization, was used). For example, it is feasible to increase the radiance level from five to 10, or even 20 in lab calibration. However, it is impractical to increase the radiance level to 100 or more as it requires a large number of radiation sources where (1) each source has spectral radiation stability and a distinct radiation difference from the others; (2) a combination of multiple radiation sources and many integral spheres (or reference boards) with different reflective properties; or (3) a radiation source which can fine tune the radiation energy. None of these can easily achieved. Even if the required radiance level is met, the workload of the measurements is huge because every time the radiation source is adjusted, the measurement and the record need to be done accordingly, and as a result the workload in processing the data is also increased.

However, utilizing Equations (14) and (15), the precision of the calibration coefficients can be improved rapidly via the increase of the number of measurements at each radiation level. When the number of grouping is four, the increase of the radiance level achieves the same effect as the increase of the number of measurements. In other cases, the increase of the number of measurements demonstrated a stronger effect than the increase of the radiance level. To increase the number of measurements is relatively easier to implement and does not actually add any difficulty to the whole calibration process, thus making the experiment as well as the data processing process simple and efficient. It was also noticed that this method of improving the calibration precision by increasing the number of measurements is based on the assumptions that the experimental environment and the sensors' working status are stable and the characteristics of the stochastic noise are unchanging. As the number of measurements cannot be infinitely increased, dozens of measurements should suffice. Therefore, in order to improve the precision of the relative radiometric calibration, the strategy is to increase the number of the radiance level and the number of measurements simultaneously and take the latter as the main force.

## 5. Conclusions

Based on the theoretical analysis and the computer simulation, the optimal strategy in conducting the relative radiometric calibration can be summarized as follows: (1) when the number of the radiance level and the number of measurements are fixed, the highest precision can be achieved with the error being $2\sqrt{2}M_{n}/\sqrt{IT}$ if the measurement data is evenly divided into four groups and the average is done within each group; (2) the bigger the number of the radiance level, the bigger the number of measurements at each level, therefore, the higher the precision of the calibration. When the grouping is done at four, the number of the radiance level and the number of measurements take the same effect on the precision and show a stronger impact than the other even-division ways. When no grouping is done, the number of measurements demonstrates a stronger impact than the number of radiance level; and (3) when trying to improve the calibration precision by increasing the number of measurements, the suggested number is no less than 20 but not too large, with the key to ensure the stability of the experiment environment. Therefore, the average measurement should be done within a few minutes in the lab calibration or within a few seconds in the field measurements.

## Figures and Tables

**Table 1:** The parameters used for simulations.

	*K*	*I*	*T*	*J*
Case 1	100	20	1–1000	*J* = *I*
Case 2	100	3–100	20	*J* = *I*
Case 3	100	3000	20	3–3000
